# 6-Week Supplementation with *Tribulus terrestris* L. to Trained Male CrossFit^®^ Athletes on Muscle, Inflammation, and Antioxidant Biomarkers: A Randomized, Single-Blind, Placebo-Controlled Trial

**DOI:** 10.3390/ijerph192316158

**Published:** 2022-12-02

**Authors:** Diego Fernández-Lázaro, Jesús Seco-Calvo, Jorge Pascual-Fernández, Carlos Domínguez-Ortega, Miguel Del Valle Soto, Juan Mielgo-Ayuso

**Affiliations:** 1 Department of Cellular Biology, Genetics, Histology and Pharmacology, Faculty of Health Sciences, University of Valladolid, Campus of Soria, 42003 Soria, Spain; 2 Neurobiology Research Group, Faculty of Medicine, University of Valladolid, 47005 Valladolid, Spain; 3 Physiotherapy Department, Institute of Biomedicine (IBIOMED), Campus of Vegazana, University of Leon, 24071 Leon, Spain; 4 Psychology Department, Faculty of Medicine, Basque Country University, 48900 Leioa, Spain; 5 Medical Hospital Emergency Service of Hospital San Pedro, Rioja Health, 26006 Logroño, Spain; 6 Hematology Service of Santa Bárbara Hospital, Castile and Leon Health (SACyL), 42003 Soria, Spain; 7 Department of Cellular Morphology and Biology, Universidad de Oviedo, 33006 Oviedo, Spain; 8 Department of Health Sciences, Faculty of Health Sciences, University of Burgos, 09001 Burgos, Spain

**Keywords:** *Tribulus terrestris* L., herbal supplementation, CrossFit^®^, muscle damage, inflammation response, exercise-induced oxidative stress

## Abstract

*Tribulus terrestris* L. (*TT*) ingredients have anti-inflammatory and antioxidant activities, but their effects on exercise-induced muscle damage (EIMD) in trained athletes are uncertain. The purpose of this single-blind placebo-controlled trial, in accordance with CONSORT guidelines, was to examine the effect of 6 weeks of *TT* supplementation on muscle metabolism, inflammation biomarkers, and oxidant status. Thirty trained male CrossFit^®^ athletes were randomly assigned to be supplemented with 770 mg/day of *TT* (intervention group (IG)) or receive a placebo daily (control group (CG)) for 6 weeks. Muscle damage enzymes, inflammation biomarkers, and Total Antioxidant Status (TAS) were assessed at baseline (T1), 21 days after baseline (T2), and after 42 days (T3). Grace, a Workout of the Day, was measured in T1 and T3. Statistical significance (*p* < 0.05) was found between IG and CG in Lactate Dehydrogenase (LDH), C-reactive protein (CRP), and TAS levels at the end of the follow-up. Furthermore, TAS levels were significantly (*p* < 0.05) lower at T2 and T3 relative to baseline in the IG, also LDH and CRP increased significantly (*p* < 0.05) at T2 and T3 relative to baseline in the CG. No significant (*p* > 0.05) decreases in muscle damage or inflammation biomarkers were observed, although a slight downward trend was observed after 6 weeks for supplemented athletes. *TT* supplementation could attenuate the CrossFit^®^ training program-induced oxidative stress, muscle damage, and inflammation which could be due to the natural antioxidant and anti-inflammatory properties of *TT*.

## 1. Introduction 

CrossFit^®^ is a fitness methodology based on standardized and varied functional movements performed at high intensity called “Workouts of the Day” (WODs) [1]. It is designed to improve general fitness parameters (cardiovascular endurance, skeletal muscle strength, body composition, flexibility) and sports performance (agility, speed, power, strength, accuracy) [2]. In CrossFit^®^ training time, all WODs are completed at maximum intensity, in a repetitive way without recovery time in between [3]. In particular, the focus on constant variation of functional movements in CrossFit^®^ training allows for the use of core elements of gymnastics, weightlifting exercises, and cardiovascular activities as exercise tasks. Thus, CrossFit^®^ is also considered an option for high-intensity interval training (HIIT) [2]. Extenuating physical activity leads to tissue damage resulting from an excessive inflammatory reaction that can impair muscle performance [4]. Exercise-induced muscle damage (EIMD) triggers an inflammatory response with a decrease in muscle strength, localized swelling, delayed onset muscle soreness (DOMS), and an increase in muscle proteins in the blood such as creatine kinase (CK), lactate dehydrogenase (LDH), and myoglobin (Mb) [5,6]. Additionally, EIMD triggers inflammatory responses that result in elevations of inflammatory markers such as C-reactive protein (CRP) interleukins (IL) IL-1, IL-6, and tumor necrosis factor (TNF-α) [5,7]. Consequently, EIMD, can negatively affect an individual’s athletic performance and training quality, especially training with short recovery periods. 

The high physical demands of CrossFit^®^ means that the usual nutritional recommendations needed to improve athletes’ performance are insufficient [8,9]. Nutrition is the foundation of fitness and health for CrossFit, and it is considered the basis for WODs. Since CrossFit^®^ is a sport with one of the most demanding physical components on the planet (it employs a combination of many training disciplines), it utilizes all energy systems in strenuous WODs. Therefore, it seems reasonable to implement nutritional aids that allow the nutritional requirements of athletes to be met, optimizing their health and athletic performance, which are also consistent with the principles of rational nutrition. [8]. In this regard, the use of herbal supplements has increased in recent years due to their potential effects on health and physical performance, attributed to their bioactive components [10]. *Tribulus terrestris* L. (*TT*) is one of the most widely marketed herbal supplements among the Western population and is commonly used as a medicinal herb in Ayurvedic medicine [11]. 

*TT* is an exotic plant from the Zygophyllaceae family, genus *Tribulus*, which includes 20 different plants species. The most widely known bioactive components of *TT* are saponins, flavonoids, glycosides, alkaloids, and tannins, among others. [12]. *TT* saponins possess moderate dose-dependent anti-inflammatory properties and *TT* polyphenols, flavonoids, and act as antioxidants and effectively scavenge the free radicals in a concentration-dependent manner [12,13]. Therefore, *TT* would reduce inflammation [14] by attenuating muscle damage [15] modulating and oxidative damage [16].

Considering that athletes need to mitigate EIMD, for quick recovery between workouts or competitions, the use of well-supported nutritional and supplementation strategies can be important allies of CrossFit^®^. The aim of the present study was to evaluate the effect of 6 weeks of *TT* intake on the enzymes and proteins indicative of muscle metabolism as serum Mb, CK, LDH, Alanine Aminotransferase (ALT), Aspartate Aminotransferase (AST), and Aldolase; inflammatory biomarkers as IL-6 and CRP levels; and oxidant status as Total Antioxidant Status (TAS) levels, in trained CrossFit^®^ athletes. Additionally, we have controlled the fatigue level by “Grace” (WODs) during a high-intensity CrossFit^®^ training program. We hypothesize that *TT* would substantially modulate muscle damage, inflammation, and oxidative status induced by the CrossFit^®^ training program, without toxicity in athletes. We have previously conducted a randomized, single-blind, placebo-controlled study on CrossFit^®^ athletes, evaluating hormonal behavior, body composition, and perceived exertion [17]. Thus, with the methodological structure of our previous study [17], we have conducted a placebo-controlled trial on CrossFit^®^ athletes.

## 2. Materials and Methods

### 2.1. Participants 

Participants’ characteristics are shown in Table 1. Thirty adult men, healthy and nonsmokers, volunteered to participate in this study. Volunteers were trained and participated in regular exercise on average ≥ 3 times per week for the previous 12 months. In this study, the following inclusion criteria were established: (i) ≥20 months of previous CrossFit^®^ training; (ii) perform “Fran” (WODs) ≤ 250 s; (iii) men; (iv) age range between 18 and 50 years (both inclusive). Exclusion criteria included any kidney misfunction, a known intolerance or allergy to herbal supplements, a recent febrile illness, lower limb trauma, or any history of muscle injury. 

Participants had not taken any antioxidant, anti-inflammatory and performance, or immune function-enhancing medications or dietary supplements in the 3 months previous to the study. Participants were also asked to avoid any intensive exercise or heavy physical activity throughout the study and abstain from ingesting caffeine, alcohol, and illicit drugs that modify muscle behavior and disturb physical performance before and during the exercise trials. Before the study, all participants were informed about all possible risks or discomforts and benefits associated with the study and signed the consent form. The experiment procedures were approved by the Clinical Research Ethics Committee (CREC) of Valladolid Clinical Hospital (PI-19-1350).

### 2.2. Research Design

A randomized, single-blind, placebo-controlled trial was performed to study whether supplementation with *TT* over 6 weeks can improve or reduce muscle damage and inflammation biomarkers, following a structured CrossFit^®^ workout. All participants maintained an identical diet throughout the 6 weeks, inspected by a nutritionist on the research staff of this study. 

We have applied the guidelines of the Consolidated Standards of Reporting Trials (CONSORT) (Table S1) [18].

The sample size estimation was performed using the G* Power 3.1.97 statistical power analysis program (University of Dusseldorf, Dusseldorf, Germany; accessed on https://es.freedownloadmanager.org/Windows-PC/Gpower-GRATIS.html) (accessed on 10 July 2021) [19]. The number of mandatory participants was calculated to measure differences between independent groups, following the methodology of our recent study [20] for trials with small sample sizes. A sample of *n* = 15 participants per study group for a total of *n* = 30 volunteers was needed, considering a dropout rate ≤ 20%. At the beginning of the 6-week intervention period, individuals randomly received pre-packed capsules which included either a placebo (PL) or the *TT* supplement and labeled with the day of consumption. The randomization sequence was statistically designed to assign the participants to the two study groups (control group (CG) and intervention group (IG)) using a stratified block design. The IG received 2 *TT* capsules from Quamtrax^®^ Pharmaceuticals (Quamtrax^®^ Nutrition Europe S.L. Fuenlabrada, Madrid, Spain) with 770 mg daily; *TT* extract 72.64% (385 mg/cap) at 40% of saponins. The CG received 2 placebo capsules of 100 mg, formulated using maltodextrin capsules to simulate the color and texture of the *TT* capsules, to generate a highly reliable blinding. The placebo was manufactured at the Magistral Formulation Laboratory of a Pharmacy (Soria, Spain). Participants were instructed to ingest the capsules orally with 200 mL of water, in 1 daily dosage, taken 30 min after CrossFit^®^ workout.

### 2.3. Dietary Assessment

To standardize diet and antioxidant status, participants were advised to have the same standard diet (50% carbs, 40% protein, and 10% fat) prescribed by a registered professional dietitian-nutritionist. This dietitian-nutritionist carefully documented in detail the daily food and fluid intake of the subjects throughout the trial to ensure that they had followed the diet they were given. Participants were instructed on how to complete the diet-recall questionnaires and determine food servings and sizes by a registered professional dietitian. The CrossFit^®^ athletes were instructed using 2 diet tracking methods previously used in our studies [17,21]: (i) a food frequency questionnaire (FFQ) that included 139 different foods and beverages, sorted by food type and meal pattern, at T3 of the previous 6 weeks, and (ii) a 7-day dietary recall questionnaire of the 7 days prior to T3. The data obtained from each food were converted into micronutrients, macronutrients, and total energy intake, using the standardized Easy diet© software. Additionally, we proceeded to measure the total energy intake * Kg^−1^ for each CrossFit^®^ athlete [22].

### 2.4. Anthropometric Measurements

An internationally certified anthropometrist (ISAK level 3 with certificate number: #63673929292503670742) performed the anthropometric measurements of all CrossFit^®^ athletes. Bioelectrical impedance analysis (BIA) (BC-730; Tanita, Japan) was employed to calculate body mass and fat mass; a stadiometer was used to measure height. 

### 2.5. Blood Collection and Biomarkers Analysis

**Figure 1:**
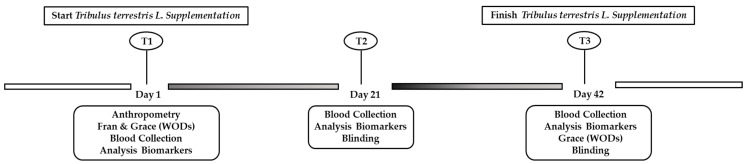
The implementation schedule for the 6 weeks of the study.

All participants attended the laboratory for blood collection at 3 specific times: at baseline (T1), 21 days after baseline (T2), and at the end of the study after 42 days (T3) (Figure 1). Blood samples (10 mL each) were collected from the antecubital vein of all athletes at each point (T1, T2, and T3), under baseline conditions, after an overnight fast, and 36 h without exercise. For blood collection, CrossFit^®^ athletes arrived at the laboratory at 8:30 am and upon arrival sat comfortably for 30 min. The collection and transport of blood samples was performed following the World Anti-Doping Agency (WADA) standards [23]. The preparation of the blood samples prior to the determination of the biomarkers was performed following our previously described methodology [17,21]. Serum myoglobin (Mb) was determined by a chemiluminescent luminol reaction after adsorption to anti-myoglobin IgG onto a solid phase. %CK-MB fraction, calculated as (CK-MB/CK) * 100, LDH, aldolase, ALT /GPT, and GOT/AST were measured using classic enzymatic methods, in a Hitachi autoanalyzer (Hitachi 917, Japan). Urea was measured using a colorimetric enzymatic method and creatinine was measured using the Jaffe’s reaction adapted kinetic method. Human IL-6 ELISA™ kit (ab178013) (INVITROGEN; ThermoFisher Scientific, Waltham, Massachusetts, United States) was used to measure the serum IL-6 level with a sensitivity of <2 pg/mL. CRP was also measured with the ELISA method using a CRP Human ELISA™ kit (BMS288INST) (INVITROGEN; ThermoFisher Scientific, Waltham, Massachusetts, United States) with a sensitivity of <10 pg/mL. The white blood cells (WB), monocytes (MON), lymphocyte (LIM), red blood cells (RCB), hemoglobin (Hb), and hematocrit (Hct) were evaluated in a System Coulter Counter MAX-M model hematological counter. TAS was determined by an automated Rel Assay measurement method Total Antioxidant Status (E-BC-K801-M) (Elabscience Biotechnology, USA). TAS is usually used to estimate the overall oxidation state of the body [24]. All biomarker tests were performed in a public hospital of the Castile and Leon Health Network (SACyL), Santa Barbara Hospital of Soria (Spain), with the corresponding controls of the technique. Percent variation in plasma volume (% ΔPV) was estimated using Van Beaumont’s method. Moreover, analytical marker values make an adjustment by: Corrected value = Uncorrected value * ((100 + % ΔPV)/100) [22].

### 2.6. CrossFit Training

Participants trained in 3 sessions on alternate weekdays (Monday, Wednesday, and Friday) for a total of 4.5 h/week. The start of all CrossFit^®^ training sessions was between 6:00 p.m. and 8:00 p.m. Each 1.5 h CrossFit^®^ training session consisted of 3 sections such as specific warm-up, WODs, and cool-down. Each workout was performed in the participants’ own gym, supervised by a certified CrossFit^®^ Degree 1- or 2-certified instructor (each). All subjects completed the same physical activity routines to ensure that they performed the same prescribed workout during the study. In addition, CrossFit^®^ athletes were specifically asked not to perform CrossFit routines outside of the structured training program.

### 2.7. “Workouts of the Day” (WODs)

Fran’s inclusion WODs consisted of three rounds of thrusters and pull-ups for 21, 15, and 9 repetitions [1,2]. Fran is one of the most famous types of training that all CrossFit^®^ athletes do to monitor their performance improvements. Thanks to its great scalability, Fran has become famous as a key exercise to know the athlete’s progress and many CrossFit^®^ athletes use it as a unique yardstick to measure their physical capacity and analyze physical progress [25]. Furthermore, Zeitz et al. [26] have reported that the 33% increase in performance variation in Fran can be explained by improvements in strength. Butcher et al. [27] verified that Fran was strongly correlated with strength data and oxygen consumption at the anaerobic threshold. The 250 s mark is the cut-off point for participation in local amateur CrossFit^®^ competitions, and it is considered an advanced level of CrossFit^®^ with times over 4 min [1,2].

We have controlled the fatigue level using the Grace test (WODs) following the international CrossFit^®^ protocols [1,2]. For Grace, CrossFit^®^ athletes performed 30 clean and jerk repetitions, and the time in seconds (s) to complete them was measured. The male athletes were required to use weights of 61.4 kg [1,2]. The Grace test was assessed at baseline (T1), when *TT* supplementation had not yet begun, and at the final day of the study (T3) after 6 weeks. 

### 2.8. Blinding 

The participants (*n* = 30) completed a form intended to assess the effectiveness of the single-blinding process in the study. The results of the questionnaire indicated that 90% (*n* = 27) of the CrossFit^®^ athletes did not identify the group to which they were assigned. Only 1 participant stated and correctly knew which group in the study he belonged to. These results could indicate that the single blinding was fruitful. 

### 2.9. Data Analysis

For the random assignment of participants to both study groups (CG and IG), we used the sequence created by the “Random Sequence Generator” software (based on stratified block design) based on study entry date (available at https://apps.apple.com/es/app/random-number-generator-app/id1476396989) (accessed on 15 July 2021). The statistical analysis was carried out using STATA version 15.0 (StataCorp, College Station, TX, USA), SPSS software version 24.0 (SPSS, Inc., Chicago, IL, USA), and Microsoft Excel (Microsoft Excel version 19). The results were contained as means and standard deviations. Statistical significance was established at a value of *p* < 0.05. The Shapiro–Wilk test was used to determine the normality of the variables. Parametric tests were used because the data followed a normal distribution. To determine the differences between the groups (IG and CG) at baseline and the end of the study, a t-test for independent variables was used for the sample characteristics, dietary assessments, and performance tests. In addition, a t-test for dependent variables was used to determine the existence of significant differences between the performance tests at T1 and T3. An analysis of variance (ANOVA) with repeated measures was performed to examine the effects of the time × *TT* supplementation interaction on the different groups (IG and CG) and the muscle damage (AST, ALT, CK, CK-MB, LDH, and Mb), inflammation (IL-6 and CRP), and oxidant response (TAS) of the groups. The differences between the biomarkers at different times during the study were determined using the Scheffé test. The percent changes in the WODs test studied in the CG and IG between T1 and T3 were calculated through the following formula: Δ% = ((T3 − T1)/T1) ∗ 100; to determine whether the differences in these percentages between groups were significant, the *t*-test for independent samples was used.

## 3. Results 

### 3.1. Recruitment and Randomization 

Forty-five CrossFit^®^ athletes were enrolled; however, seven participants declined to take part in the study. The remaining 38 CrossFit^®^ athletes were tested with “Fran” (WODs), and 6 athletes were discarded for presenting results, time to complete the test > 250 s. In addition, two participants were rejected because they chronically consumed anti-inflammatory drugs that could alter the biomarkers of the study. Finally, 30 CrossFit^®^ athletes were involved in the study. Thirty athletes were randomly assigned into two groups (control group (CG; *n* = 15) and intervention group (IG; *n* = 15)). None of the 30 participants dropped out of the study or interrupted the intervention, so all participants were tested at T1, T2, and T3 (Figure S1).

No significant differences (*p* > 0.05) were obtained in the anthropometric and exercise performance profile between CG and IG, as described in Table 1.

### 3.2. Dietary Assessment

During the study, there was no significant difference between CG and IG (*p* > 0.05) for the dietary components reported in Table S2. 

### 3.3. Blood Collection and Biomarkers Analysis

The % ΔVP of the CrossFit^®^ athletes was reduced by 3.3 % between baseline and T2. In addition, % ΔVP also decreased by 3.7% between baseline and T3. Following these results, we proceeded to the adjustment of all biomarkers.

### 3.4. Muscle Biomarkers

No significant differences (*p* > 0.05) between the time points (T1, T2, and T3) nor between CG and IG were detected for ALT, AST, Mb, aldolase, CK, CK-MB. Significant differences (*p* < 0.05) were observed between the three study points in the CG for LDH. In addition, for LDH there were significant differences (*p* < 0.05) in *Condition × Time* between both groups (CG and IG). For the CG, a significant increase (*p* < 0.05) in LDH was observed at T2 (353.11 ± 9.12 UI/L) and T3 (366.50 ± 10.71) compared to T1 (312.13 ± 10.81 UI/L). However, LDH had a downward trend in the IG from baseline (328.59 ± 5.49 UI/L) to T3 (314.14 ± 4.70 UI/L) (Table 2).

### 3.5. Inflammatory Biomarkers

Significant differences (*p* < 0.05) were observed between the three study points (T1, T2, and T3) in the CG for CRP. In addition, for CRP there were significant differences (*p* < 0.05) in *Condition × Time* between both groups (CG and IG). In addition, for the CG, a significant increase (*p* < 0.05) in CRP was observed at T2 (1.51 ± 0.22 mg/L) and T3 (1.55 ± 0.22 mg/L) compared to T1 (1.21 ± 0.30 mg/L) (Table 2). However, in the IG a slight non-significant (*p* >0.05) decrease was observed throughout the three points evaluated in the study (T1, T2, and T3) (Table 2).

### 3.6. Oxidant Status

For the IG, TAS was significantly increased (*p* < 0.05) at T2 (1.46 ± 0.02 mmol/L) and T3 (1.48 ± 0.05 mmol/L) compared to T1 (1.34 ± 0.07 mmol/L). In the CG, TAS remained practically unchanged from T1 (1.31 ± 0.05) to T3 (1.33 ± 0.06). Significant differences (*p* < 0.05) between both groups, IG and CG, were observed for TAS (Table 2).

### 3.7. Hematological Biomarkers 

No significant differences (*p* > 0.05) between the time points nor between the groups were reported for hematological biomarkers (Table 3).

### 3.8. Workouts of the Day (WODs)

Table 4 indicates the percentage changes (%Δ) on the Grace test. There were no significant differences (*p* > 0.05) in Grace, but there was a slight trend of improvement in the IG (IG −25.00 ± 10.74 %Δ) compared to the CG (−24.73 ± 10.58%Δ).

## 4. Discussion

Our study’s results showed that LDH, CRP, and TAS levels after 6 weeks of supplementation with *TT* (770 mg/day) in trained male CrossFit^®^ athletes were significantly (*p* < 0.05) different compared to CG, throughout the three points of the study. In addition, TAS levels were significantly (*p* < 0.05) lower at T2 and T3 relative to baseline in the IG. No significant (*p* > 0.05) decreases in muscle damage or inflammation biomarkers were observed, although a slight downward trend was observed after 6 weeks of the study for supplemented athletes. For a better understanding, we have divided the discussion into several sections.

### 4.1. Inflammatory Biomarkers

WODs are recognized as one of the modalities of very-high-intensity functional training with minimal or no rest between bouts, making WODs a strenuous workout [3]. In response to demanding physical exercise, proinflammatory mediators are released, such as IL-6, IL-1, IL-4, or TNF-α and acute phase proteins such as CRP, increasing their plasma levels [28]. These mediators are biomarkers of the inflammatory response [29]. In a previous study [15], *TT’s* supplementation for 2 weeks with 500 mg/day had no effect on IL-6 or hs-CRP in healthy non-athletic men after resistance exercise. According to these findings, we did not observe any effect on IL-6. However, we showed a significant difference regarding between-group changes in CRP (*p* < 0.05) and a significant (*p* < 0.05) increase in the CG, with a moderate downward trend in the IG. These differences could be due to the supplementation time, dose, or composition of *TT´s* supplement.

The potential anti-inflammatory effect of *Tribulus* may be due to the modulating action on inflammatory pathways of *TT* components [30]. The polyphenols could act by inhibiting NF-Bκ activation, suppressing the activation and phosphorylation of JAK/STAT proteins [31,32], but the flavonoids would also act by inhibiting COX-2 and prostaglandin E2 (PGE2) production by decreasing the expression of p-JNK [31]. Based on the above mechanisms [31,32], it can be hypothesized that the 6-week consumption of TT may be effective in controlling the overall inflammatory state by modulating CRP increases. However, TT supplementation in physically active individuals would not modulate the exercise-induced IL-6 response. Perhaps *TT* lacks immunomodulatory properties, which regulate inflammatory states for a longer duration. However, an in vitro study in RAW 264.7 cells [32] reported that *Tribulusamide D* (phenolic amides from the fruits of *TT*) treatment reduced the expression of inflammatory cytokines IL-6, IL-10, and TNF-α [32]. 

### 4.2. Antioxidant Biomarkers

A recent study [33] showed a significant (*p* < 0.05) decrease in plasma total antioxidant capacity after vigorous WODs. A CrossFit^®^-based metabolic conditioning session increases oxidative stress in proportion to the intensity or fatigue induced by the duration of the training session [34]. The high metabolic and mechanical demands of CrossFit^®^ would trigger different major sources of reactive oxygen species (ROS) contribution during and after WODs [33]. *TT´s* polyphenols and flavonoids act as antioxidants [12,35] which could modulate oxidative damage [16] by neutralizing the ROS, and potentially enhance post-WODs recovery [36]. Da silva et al. [37] reported the decrease of oxidative stress in endurance training athletes supplemented for 8 weeks with 500 mg/day of *TT*. In addition, in cadmium-intoxicated rats supplemented with *TT* extract (5 mg/kg) that reduced the elevation of thiobarbituric acid reactive substances (TBARS) [37], thiobarbituric acid was formed as a by-product of lipid peroxidation. These results agree with those reported in our study, where IG significantly (*p* < 0.05) increased TAS. TAS evaluates the dynamic balance between the antioxidant system and prooxidants [6]. Regarding the antioxidant activity of *TT*, we could hypothesize that long-term supplementation of *TT* (6 weeks, 770 mg/day) could be effective in increasing TAS in the CrossFit^®^ training program.

### 4.3. Muscle Biomarkers

After a high-intensity CrossFit^®^ training program, a process of muscle tissue breakdown and dumping of intramyocellular contents into the systemic circulation is initiated [38]. Sustained and intense WODs can initiate a significant elevation of several plasma stress mediators as markers of circulating muscle damage [33]. These biomarker elevations are indicative of increased EIMD [5], which negatively influences the performance and/or health of athletes [29]. EIMD increases muscle enzyme activity [21]; similarly, CG non-supplemented athletes showed increased muscle enzyme activity, with serum LDH levels being significantly higher at T2 and T3 than baseline. However, muscle damage biomarkers showed a slight downward trend in *TT* supplemented athletes, especially on LDH plasma levels, where their behavior was statistically significant (*p* < 0.05) compared to the non-supplemented. Overall, muscle biomarker levels of the participants in the IG were lower than those of the CG after the exercise, but this decrease was not statistically significant. Supplementation of *TT* at higher doses or for a longer period could lead to a significant improvement. Three previous studies examined muscle damage biomarkers [15,39,40] in physically active adult males. In these studies, *TT* supplementation demonstrated a significant reduction for CK activity (during high-intensity training) [39] and a non-significant decrease [15]. In addition, LDH activity (during resistance-exercise training) was significantly reduced after the administration of 500 mg/day of *TT* powder and extract [15]. However, Milasius et al. [40] found significant increases in CK plasma levels in male endurance athletes supplemented with *TT*. These differences may be due to bioactive components of *TT* supplementation [41]. *TT´s* muscle protective action via flavonoids, alkaloids, phenols, and saponins [15,39] are necessary to have an antioxidant and/or anti-inflammatory effect [11,42]. Therefore, in tissue injury skeletal muscle, membrane permeability would be reduced, thereby reducing the release of muscle-damaging enzymes from skeletal muscle into the blood circulation [41]. Therefore, such results could suggest that *TT* might effectively lessen the muscle damage because of a CrossFit^®^ intense training-based regimen. 

### 4.4. Tribulus terrestris L. Supplementation 

Human clinical trials reported several side effects after *TT* treatment such as: gastrointestinal problems [43,44], gynecomastia [45], priapism [46], nephrotoxicity [47,48], hepatotoxicity, neurotoxicity [47], sleep disturbances, fatigue, hypertension, and elevated heart rate [35,48]. Our athletes have not expressed adverse effects during or after the 6-week supplementation protocol with *TT* (770 mg/day), and no alterations on hematological biomarkers have been reported. The *TT´s* dose administered was within the safety range (250 mg–9000 mg) proposed by the Scientific Committee of the Spanish Agency for Consumer Affairs, Food Safety and Nutrition (AECOSAN) [49].

### 4.5. CrossFit^®^ Training Program

A CrossFit^®^ training program was performed looking for athletes to perform movements with the highest possible intensity to generate fatigue and induce a certain degree of muscle damage and oxidative stress [50,51]. Our CrossFit^®^ training program performed with athletes suggests that the results could be considered accurate, as the evaluated results showed alterations in muscle metabolism and biomarkers of inflammation and oxidative status. In addition, the CrossFit^®^ training program increased athletic performance in the Grace test, which could imply suitability as a physical training program. In addition, prolonged periods of intensive exercise training (physical stress conditions) can reduce immunity and decrease red blood cells, hematocrit, and hemoglobin levels [20]. In our study, no differences were found in hematological biomarkers. Thus, our CrossFit^®^ training program and 6-week supplementation regimen with TT (770 mg/day) could be appropriate. 

#### Limitations and Strengths

The main limitation was the small sample size, which limits the interpretation of the results. Gender may be a factor influencing the severity of muscle damage, oxidative stress, and inflammation responses in humans (only male athletes were included). In addition, the percentage of components of the *TT* supplement administered was not determined. 

On the other hand, the strengths of our study were the randomized single-blind placebo-controlled design, the application of CONSORT guidelines, the inclusion of athletic subjects with previous CrossFit^®^ experience, no differences between the two study groups (IG and CG), and the control of dietary intake. 

## 5. Conclusions

Oral *TT* supplementation of 770 mg/day in CrossFit^®^ trained men could slightly attenuate the exercise-induced oxidative stress, muscle damage, and inflammation which could be due to the natural antioxidant and anti-inflammatory properties of *TT*. Therefore, further studies are needed in this field to corroborate the effects of *TT* on muscle performance/behavior in athletes training at a high intensity.

### Practical Applications

*TT* supplementation could be used during seasons of high physical demand, to intensify skeletal muscle recovery and counteract oxidative damage and inflammation generated by EIMD. It is necessary to consider that the supplementation strategy implemented on athletes should be done considering the physiological effects of *TT*.

## Figures and Tables

**Table 1:** Participant’s Anthropometric and Exercise Performance Profile.

	CG (*n* = 15)	IG (*n* = 15)	*p*-Value
**Age (years)**	32.9 ± 6.3	33.1 ± 5.7	0.126
**Height (m)**	1.74 ± 3.3	175.1 ± 2.7	0.318
**Body Mass (kg)**	81.2 ± 11.5	80.1 ± 10.7	0.254
**Fat Mass (kg)**	10.6 ± 2.1	11.1 ± 1.9	0.152
**Fran WOD (seconds)**	233 ± 12	229 ± 14	0.345
**CrossFit^®^ experience**	41.3 ± 17.5	42.3 ± 18.3	0.176

Data are expressed as mean standard deviation. *p*: Differences between groups using one-way ANOVA. IG: intervention group; CG: control group.

**Table 2:** Muscle damage enzymes, inflammation biomarkers, and antioxidant status in the intervention group (IG *n* = 15) and in the control group (CG *n* = 15), in the three moments of the study.

Parameter (Reference Value)	Group	Time			*p*_1_-Value	*p*_2_-Value
		T1	T2	T3		
**Alanine Aminotransferase ** **UI/L (10–50)**	**IG**	23.91 ± 1.82	24.12 ± 2.30	24.12 ± 2.30	0.279	0.131
	**CG**	23.61 ± 1.44	23.47 ± 1.18	23.80 ± 1.71	0.324	
**Aspartate Aminotransferase UI/L (10–50)**	**IG**	31.21 ± 3.25	30.95 ± 3.05	30.01 ± 3.11	0.274	0.237
	**CG**	30.81 ± 2.83	31.34 ± 4.08	31.71 ± 3.46	0.235	
**Myoglobin** **ng/mL (25–72)**	**IG**	31.14 ± 2.1	30.89 ± 2.07	30.07 ± 2.88	0.224	0.177
	**CG**	31.51 ± 2.54	31.81 ± 3.04	32.36 ± 2.96	0.101	
**Aldolase ** **UI/L (1.0–7.5)**	**IG**	3.32 ± 0.33	3.31 ± 0.34	3.28 ± 0.41	0.354	0.106
	**CG**	3.28 ± 0.40	3.32 ± 0.49	3.37 ± 0.33	0.549	
**Lactate Dehydrogenase** **UI/L (135–250)**	**IG**	328.59 ± 5.49	322.07 ± 7.17	314.14 ± 4.70	0.234	0.024
	**CG**	312.13 ± 10.81	353.11 ± 9.12 ^&^	366.50 ± 10.71 ^&^	0.038	
**Creatine Kinase** **UI/L (0–190)**	**IG**	211.74 ± 3.74	203.07 ± 4.57	198.07 ± 6.03	0.234	0.133
	**CG**	200.21 ± 4.6	208.90 ± 4.57	215.77 ± 5.18	0.127	
**Creatine Kinase-MB** **<12 UI/l**	**IG**	12.30 ± 1.81	12.11 ± 1.16	12.06 ± 1.29	0.129	0.278
	**CG**	13.41 ± 1.11	13.90 ± 1.31	14.04 ± 1.33	0.236	
**Interleukin 6 ** **pg/mL (0.6–2.4)**	**IG**	0.53 ± 0.13	0.54 ± 0.16	0.52 ± 0.12	0.364	0.301
	**CG**	0.51 ± 0.12	0.53 ± 0.11	0.54 ± 0.12	0.157	
**C-reactive protein ** **mg/L (0.0–5.0)**	**IG**	1.15 ± 0.24	1.11 ± 0.31	0.98 ± 0.39	0.129	0.031
	**CG**	1.21 ± 0.30	1.51 ± 0.22 ^&^	1.55 ± 0.22 ^&^	0.033	
**Total Antioxidant Status (mmol/L) (0.0–8.0)**	**IG**	1.42 ± 0.07	1.36 ± 0. 02 ^&^	1.34 ± 0.05 ^&^	0.041	0.046
	**CG**	1.31 ± 0.05	1.34 ± 0.07	1.33 ± 0.06	0.316	

Data are expressed as mean ± standard deviation. *p*_1_-value: analysis of variance (ANOVA) with repeated measures for each group separately. *p*_2_-value: analysis of variance (ANOVA) with repeated measures of two factors to verify the existence of an interaction effect (Condition × Time). Significant differences were considered for *p* < 0.05. Significant differences during the study period, calculated using the Scheffé test. &: significant difference vs. T1, *p* < 0.05. IG: intervention group; CG: control group; T1: baseline; T2: day 21; T3: day 42.

**Table 3:** Hematological biomarkers in the intervention group (IG *n* = 15) and in the control group (CG *n* = 15), in the three moments of the study.

Parameter (Reference Value)	Group	Time			*p*_1_-Value	*p*_2_-Value
		T1	T2	T3		
**White Blood Cells** **∗10^3^/µL (3.8–11)**	**IG**	4.71 ± 0.97	4.68 ± 0.97	4.61 ± 1.04	0.774	0.187
	**CG**	4.90 ± 1.50	5.0 ± 1.46	4.76± 1.34	0.235	
**Monocytes ** **(%) (2.5–10)**	**IG**	6.72 ± 1.01	6.46 ± 1.21	6.47 ± 1.21	0.136	0.197
	**CG**	6.56 ± 1.67	6.44 ± 1. 13	6.54 ± 2.21	0.409	
**Lymphocytes ** **(%) (20–51)**	**IG**	30.11 ± 6.14	30.31 ± 9.06	31.01 ± 6. 61	0.104	0.097
	**CG**	30.51 ± 7.60	30.35 ± 7.05	31.98 ± 5.91	0.401	
**Red Blood Cells** **∗10^6^ mL^−1^ (4.5–5.7)**	**IG**	5.23 ± 0.13	5.26 ± 0.34	5.28 ± 0.21	0.314	0.188
	**CG**	5.17 ± 0.60	5.16 ± 0.49	5.18 ± 0.32	0.219	
**Hemoglobin** **g∗dL^−1^ (13–17)**	**IG**	15.21 ± 1.11	15.23 ± 0.74	15.19 ± 0.65	0.975	0.147
	**CG**	15.43 ± 0.71	15.46 ± 0.75	15.47 ± 1.93	0.892	
**Hematocrit ** **% (40–50)**	**IG**	44.17 ± 3.79	46.00 ± 3.83	46.14 ± 3.62	0.204	0.165
	**CG**	45.01 ± 2.46	45.78 ± 2.65	45.61 ± 2.591	0. 127	

Data are expressed as mean ± standard deviation. *p*_1_: analysis of variance (ANOVA) with repeated measures for each group separately. *p*_2_: analysis of variance (ANOVA) with repeated measures of two factors to verify the existence of an interaction effect (Condition × Time). Significant differences were considered for *p* < 0.05. Significant differences during the study period, calculated using the Scheffé test. IG: intervention group; CG: control group; T1: baseline; T2: day 21; T3: day 42.

**Table 4:** Percentage changes during the study on Workouts of the Day (WODs) in the two study groups: control group (CG) and intervention group (IG). Reproduced with appropriate permissions from Ref. [17]. Copyright 2021 Fernández-Lázaro, Diego. https://www.mdpi.com/2072-6643/13/11/3969 (accessed on 10 August 2022).

	Test	CG	IG	*p*-Value
**Grace Test (s)**		−24.73 ± 10.58	−25.00 ± 10.74	0.966

Percentage changes during the study. Data are expressed as mean ± standard deviation. Δ: ((T2–T1)/T1) × 100; differences among groups in each test by ANOVA test (*p* < 0.05): regarding CG. Control group: CG; intervention group: IG; Kilograms: kg; Workouts of the Day: WODs.

## Data Availability

Not applicable.

## Supplementary Materials

The following supporting information can be downloaded at: https://www.mdpi.com/article/10.3390/ijerph192316158/s1, Figure S1: A flow chart of the enrolment and randomization process, according to the CONSORT guideless; Table S1: CONSORT 2010 checklist of information to include when reporting a randomized trial; Table S2: Energy and micronutrient intake in the control group (CG) and intervention group (IG) during 6 weeks of study.
